# The Mini-Cross Prefenestration for Endovascular Repair of Aortic Arch Pathologies

**DOI:** 10.3389/fcvm.2021.745871

**Published:** 2022-01-11

**Authors:** Yifei Pei, Hongqiao Zhu, Yu Xiao, Jian Zhou, Zaiping Jing

**Affiliations:** Department of Vascular Surgery, The First Affiliated Hospital of the Navy Medical University, Shanghai, China

**Keywords:** thoracic aortic aneurysm, thoracic aortic dissection, branch artery, *in vitro* fenestration, thoracic endovascular aortic repair, aortic arch pathologies

## Abstract

**Objective:** To examine the feasibility, integrity, efficacy, and safety of endovascular repair of the aortic arch pathologies with the mini-cross prefenestration (MCPF) on stent grafts.

**Methods:** First, to prove the feasibility of the MCPF, an *in-vitro* prefenestration experiment was conducted. Second, to examine the integrity of the MCPF stent grafts, a fatigue test was conducted. Then, the membranes and metal structures of stent grafts were examined by light microscopy and scanning electron microscopy (SEM). Third, a clinical experiment was conducted to investigate the efficacy and safety of this novel technique (ClinicalTrials.gov Identifier: NCT04544579).

**Results:** All the 12 branch stents were successfully implanted and flared *in vitro*. After the fatigue test stimulating a 5-year cardiac cycle, no obvious disintegration or fracture was found in light microscopy or SEM. From December 2017 to February 2020, 26 patients with left subclavian arteries and/or left common carotid arteries involved received the novel technique. The endovascular repair with the MCPF was successfully performed on all the 26 (100%) patients. Eighteen (69.2%) patients underwent the reconstruction of the left subclavian artery (LSCA) only. The fenestrations of both the LSCA and left common carotid artery (LCCA) were conducted in 8 (30.8%) patients. Median operative time was 120 [interquartile range (IQR), 95–137.5] min and median revascularization time of the LSCA and LCCA was 30.5 (IQR, 22.8–42.0) s and 20.0 (IQR, 18.0–32.0) s separately. During the median follow-up duration of 38.9 (range, 18.8–44.2) months, one case needed an open surgery because of retrograde type A aortic dissection 3 months after implantation and no other complications or mortality occurred. The maximum aortic diameters were significantly decreased in patients with thoracic aortic dissection and thoracic aortic aneurysm (*p* < 0.05).

**Conclusion:** The existing evidence demonstrated the safety, rapid branch artery revascularization, and positive aortic remodeling of the novel technique. Long-term observation is warranted to prove the durability.

## Introduction

Thoracic aortic dissection (TAD) or thoracic aortic aneurysm (TAA) involving the aortic arch was once a restricted area of endovascular technique (1). Despite the technical advantages in thoracic endovascular aortic repair (TEVAR), completely endovascular repair of the aortic arch pathologies remains a challenge because of the insufficient landing zone and severe tortuosity (2, 3).

To simplify the open arch replacement, a hybrid technique was attempted (4). However, additional resources are required to perform such a surgery (5). With the progress of material and configuration in endovascular devices, chimney and fenestration techniques were extensively studied (6, 7). In our previous study, a single-branched stent graft was utilized to treat the aortic arch lesions and proven to have satisfactory durability and positive aortic remodeling in the long term (8). However, patients with acute aortic dissection who demand an emergency surgery may be unable to wait for this customized stent graft to be fabricated for about 2 weeks.

In this study, we asked whether the mini-cross prefenestration (MCPF) on existing stent grafts could rapidly exclude the entry tear and preserve the branch artery at the same time. To do so, we studied the preclinical and clinical applications of the novel technique. First, an *in-vitro* release test was conducted to prove the feasibility of the MCPF technique. Second, a fatigue test was conducted in fenestrated stent grafts. The integrity of main and branch stent grafts was examined by light microscopy and scanning electron microscopy (SEM). Third, a clinical experiment was approved by the Ethics Committee of Changhai Hospital, Shanghai (ClinicalTrials.gov Identifier: NCT04544579). From December 2017 to February 2020, 26 patients with aortic disease with the left subclavian artery (LSCA) and/or left common carotid artery (LCCA) involvement received the endovascular repair with the MCPF technique.

## Methods

### *In-vitro* Fenestration and Branch Stents Implantation

This part of this study was designed to assess the feasibility of the MCPF on stent grafts. The definition of successful procedure was that the delivery system of the branch stent came through the MCPF, then the branch stent was completely released, and flared with an angioplasty balloon.

**Figure 1:**
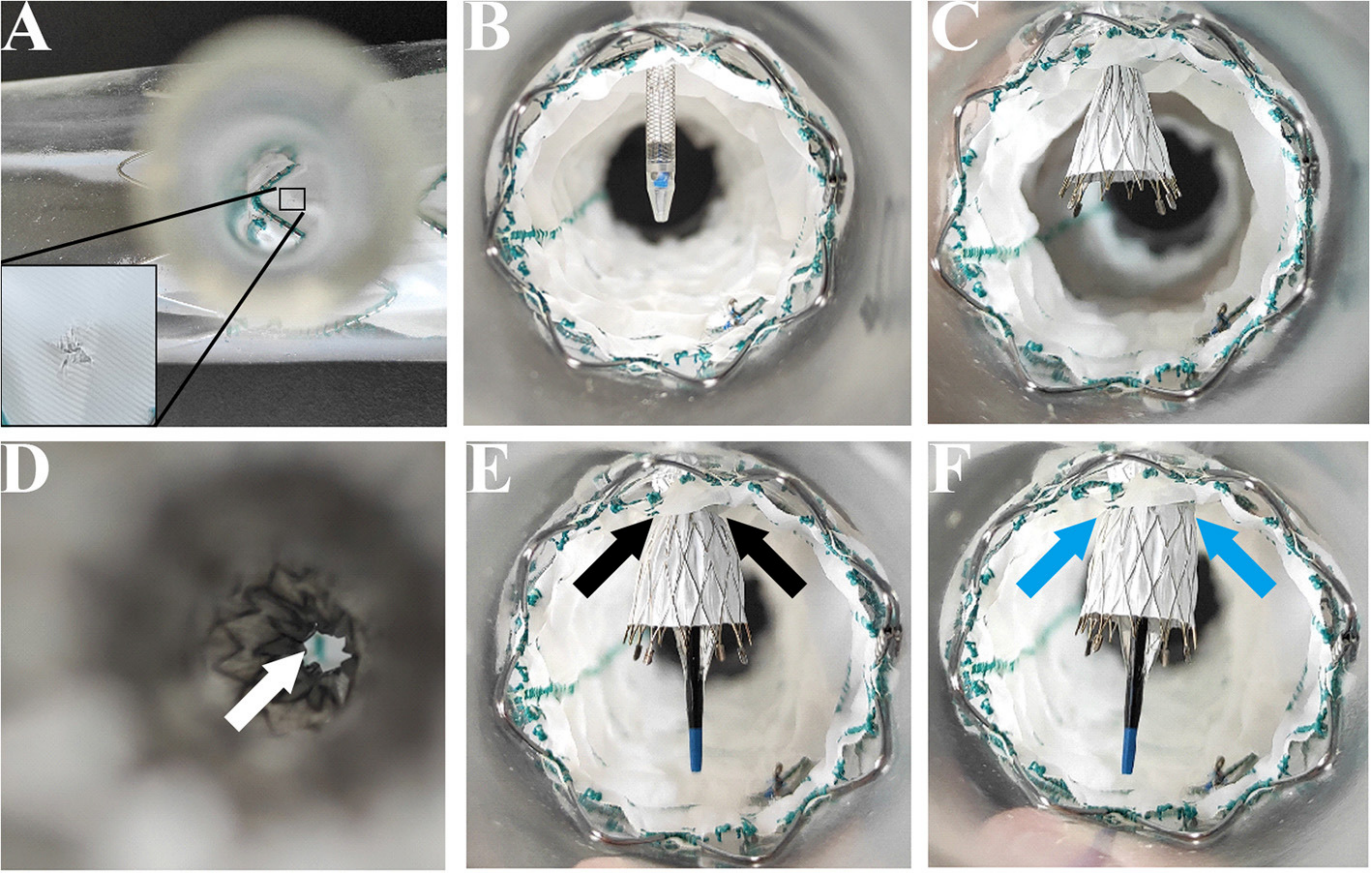
*In-vitro* experiment on the MCPF stent grafts in silicone models. **(A)** Release of the Valiant Stent Graft (diameter 34 mm and length 150 mm) with a 5 × 5 mm cross prefenestration (black square). **(B)** The hole of the fenestration is suitable to come through for an 8 Fr catheter delivery system (Fluency Plus Stent, diameter 80 mm and length 80 mm). **(C)** Release of the branch stent. **(D)** Top view of the branch stent after release. The hole was slightly expanded by the self-expanding force (white arrow). **(E)** The stent graft was flared with a peripheral angioplasty balloon at 4 atmospheres (Bard Mustang, diameter 10 mm and length 40 mm) (black arrows). **(F)** The stent graft was completely flared at 6 atmospheres (blue arrows). MCPF, mini-cross prefenestration.

The main stent grafts were 34 mm Valiant Thoracic Stent Grafts, which were generously donated by Medtronic Vascular, Santa Rosa, California, USA. All the 12 main stent grafts were released, prefenestrated with a 5 × 5 mm cross (Figure 1A), and then delivered into the silicone aortic models. There were 12 Fluency Plus Stents (Bard Peripheral Vascular, Tempe, Arizona, USA), in which the sizes were 7 × 60 mm (3 stent grafts), 8 × 40 mm (3 stent grafts), 12 × 80 mm (3 stent grafts), and 13.5 × 40 mm (3 stent grafts). The delivery system came through the fenestration (Figure 1B) and slowly released the stent grafts when tips passed 2–3 cm (Figure 1C). When the branch stents were completely released from the delivery system (Figure 1D), a 10 × 40 mm balloon (Mustang, Boston Scientific, Natick, Massachusetts, USA) was delivered to furtherly enlarge the orifice of the branch stents (Figures 1E,F).

### *In-vitro* Fatigue Test

**Figure 2:**
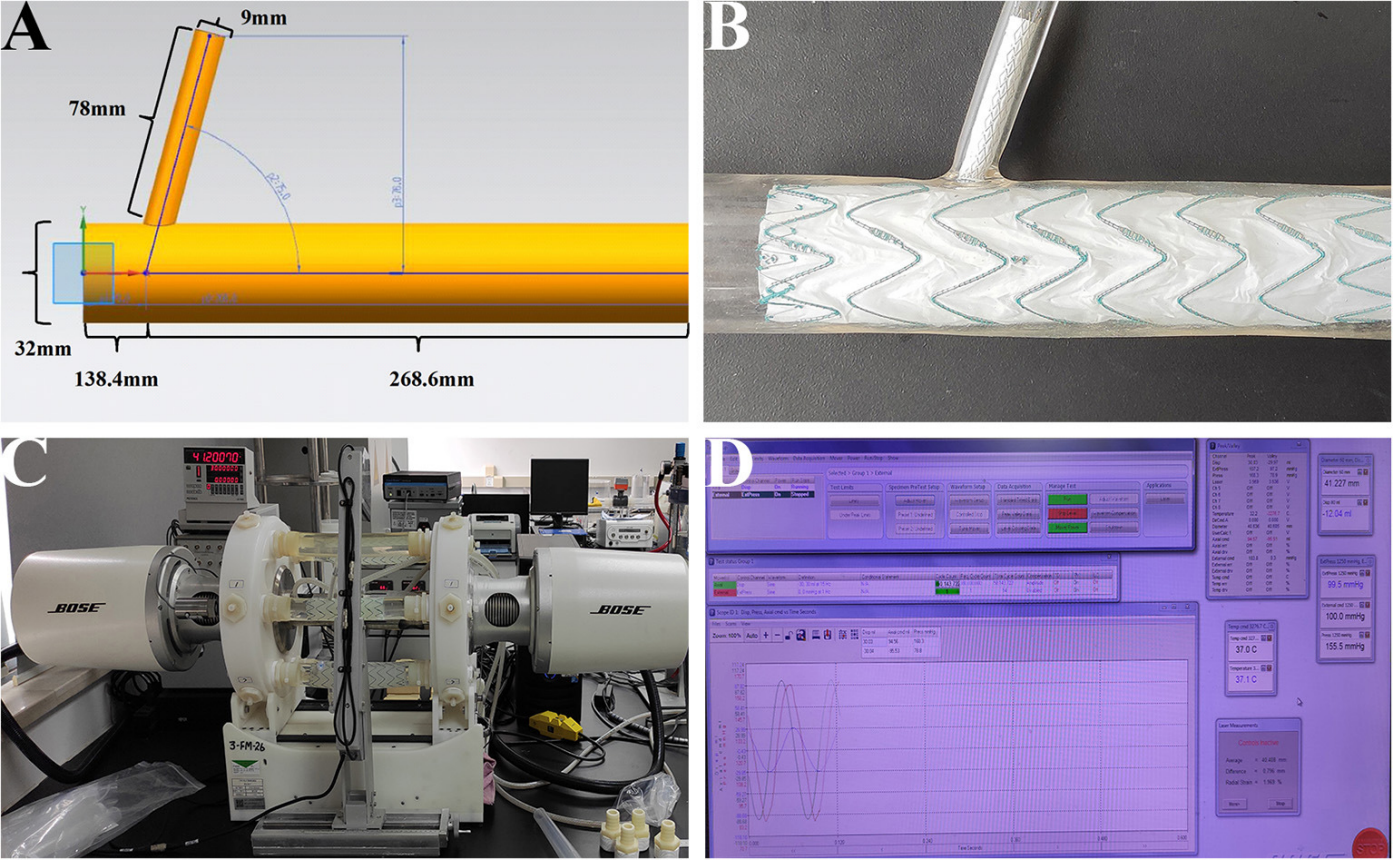
*In-vitro* fatigue experiment on the MCPF stent grafts in silicone models. **(A)** The diagram of the silicone model. **(B)** The main and branch stent grafts were implanted into the silicone model. **(C)** After stent grafts implantation, all the silicone models were installed into the fatigue test machine to stimulate the relative movement of the aorta and branch artery with the aortic pulsation. **(D)** The parameters were automatically controlled by the computer, in which the temperature was 37°C, the average systolic/diastolic water pressure was 130/80 mm Hg, and the beating rate was 1,000 bpm. MCPF, mini-cross prefenestration.

The purpose of this study was to examine whether there was disconnection, fabric breakage, or metal structure fracture on main/branch stent grafts after a fatigue test. When the stent grafts were engaged in silicone models (Figures 2A,B), the models were then installed into the fatigue test machine (Figure 2C, Bose Testing System, Minnetonka, USA). The parameters were automatically controlled by the computer (Figure 2D, WinTest Automatic Test Equipment, Yokohama, Kanagawa Prefecture, Japan), in which the average systolic/diastolic water pressure was 130/80 mm Hg and the beating rate maintained at 1,000 bpm. The solution utilized in the experiment was non-ionized water at 37°C (9). The total machine running time was 183 days to stimulate the total number of 5-year heartbeats with 100 bpm (262,800,000 cardiac cycles in all).

After the test, the silicone models were disassembled from the machine and cut along the longitudinal axis (Supplementary Figure S3A). When stent grafts were removed from silicone models (Supplementary Figure S3B), the morphologic analysis was conducted to identify any disconnection between main and branch stents by light microscopy. Furthermore, the branch stents were pulled out of the main stent grafts to detect the disconnection of fabrics beyond the fenestrations and the fractures on metal structures and membranes of branch stents. The investigation was conducted by light microscopy (Leica DM8000, Wetzlar, Germany) and SEM (SEM, EVO MA 25, Zeiss Nano Technology, Oberkochen, Germany).

### Clinical Study Population

The purpose of the clinical experiment was to investigate the safety and efficacy of this novel technique. The clinical experiment was approved by the Ethics Committee of Changhai Hospital, Shanghai (ClinicalTrials.gov Identifier: NCT04544579). All the patients and their family members signed the informed consent after being informed of the details of the procedure and potential risks.

From December 2017 to February 2020, patients with the diagnosis of aortic diseases were enrolled into this study with additional inclusion criteria, which included the LSCA and/or LCCA involvement and proximal entry tears adjacent to the LSCA (<15 mm) or the proximal seal length <15 mm (10). The exclusion criteria included the following: (1) patients who decided to receive the other endovascular techniques (18 chose Castor stent grafts, 69 chose chimney/fenestration technique, and 8 chose the LSCA partial/complete coverage during the period of study); (2) patients who were unable to tolerate general anesthesia (*n* = 3, according to experienced anesthetists); and (3) the condition that zone 1 or zone 0 was involved, which might demand a multifenestration technique (*n* = 5). Finally, 26 patients [median age, 63.0 years, IQR, 53.2–69.0; 22 (84.6%) male] with the LSCA and/or LCCA involved received the MCPF technique.

### Endovascular Procedure

**Figure 3:**
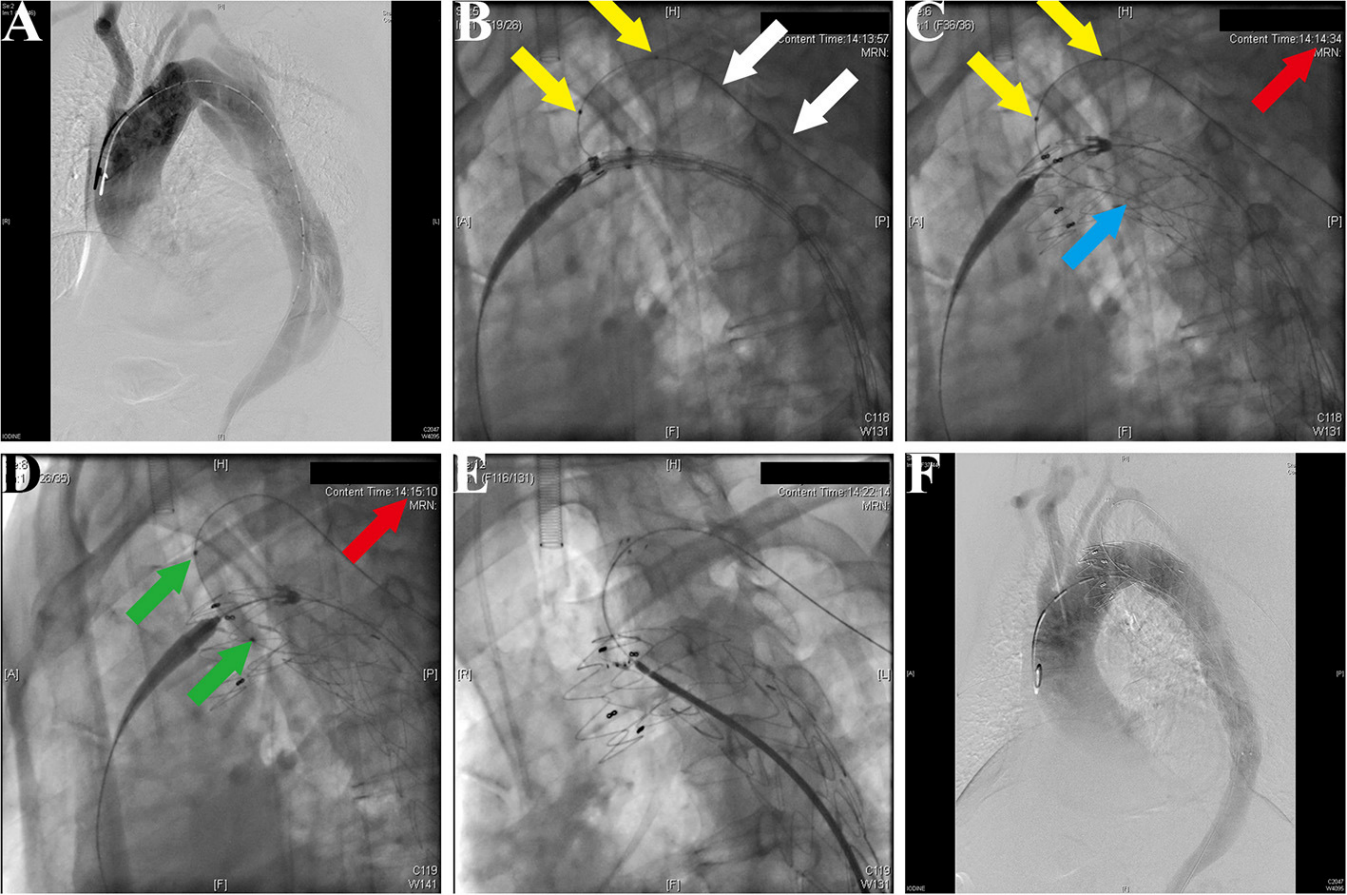
Fluoroscopic demonstration of the fenestrated stent graft delivery and engagement. **(A)** Morphological feature of aortic dissection through the digital subtraction angiography. **(B)** Delivery and advancing of the main stent graft into the proper landing zone. A traction guidewire was established through the left subclavian artery (LSCA) (white arrows). An angioplasty balloon catheter was prepared for the expansion of the fenestration (yellow arrows). **(C)** The main stent graft was released and the LSCA was temporarily covered. The traction guidewire was still in the stent graft (blue arrow). **(D)** Angioplasty of the LSCA through the guidewire from left brachial access (green arrows). The duration from coverage of the LSCA and revascularization was 36 s (red arrows, 14:14:34–14:15:10). **(E)** Deployment and engagement of the LSCA branch stent graft. **(F)** Final aortogram demonstrating patent arch branches and exclusion of the false lumen.

After general anesthesia, the left femoral artery and left brachial artery were exposed, cannulated with a 24-Fr sheath and an 8-Fr sheath separately. Through the access of the femoral artery, the digital subtraction angiography (DSA) was made by a pigtail catheter (Figure 3A). The location of prefenestration was determined by preoperative CT angiography (CTA) and intraoperative aortography. The prefenestrations were made on Valiant Captivia Stent Grafts with a 5 × 5 mm cross (Supplementary Movie 1). The range of oversizing rate was 5 to 20% according to the experience of operators. Before the stent graft was reloaded into a delivery system, the tip of a guidewire (RF^*^GA35153M, Terumo, Japan) was induced through the fenestration for the purpose of traction from the aorta to the LSCA (Supplementary Movie 2). After preparation, the traction guidewire was induced from femoral access and caught from the brachial access (Figure 3B, white arrows). Then, a peripheral angioplasty balloon catheter (Mustang, Boston Scientific, Natick, Massachusetts, USA) was induced through the guidewire in order to (1) prevent the cutting effect on the LSCA and (2) prepare for revascularization of the LSCA (Figures 3B,C, yellow arrows). When the stent graft was induced beneath the LSCA, multiple observation perspectives from DSA were adjusted to prove the traction guidewire was not twisted. The key step then was performed by one operator and two helpers. The operator controlled the delivery system and released the main stent graft; helper no.1 controlled the super stiff guidewire and helper no. 2 controlled the traction guidewire to make sure that they were in place (Figure 3C). After the release of the main stent graft, the fenestration was softly enlarged by the balloon at 6–8 atmospheres (Figure 3D) and the Fluency Plus Stent Graft was directly induced through brachial access and again flared by a peripheral angioplasty balloon at 8–10 atmospheres (Figure 3E). The branch stents were 10–20% oversized to the LSCA. When there was a need to cover LCCA for a sufficient landing zone, additional LCCA access was exposed for a traction guidewire. The choice of LCCA branch stents was according to the preference of operators, in which there were Viabahn (Gore, Flagstaff, Arizona, USA) and LifeStent (Bard Peripheral Vascular, Germany, UK) devices. The definition of technical success was the successful implantation of main and branch stents without any type I/III endoleak, migration (Figure 3F), or any other immediate major adverse cardiac and cerebrovascular events (MACCE).

### Postoperative Follow-Up

The aortic arch investigation, proximal thrombosis evaluation, and maximum descending aortic diameter measurement were performed by an experienced radiologist with the Brilliance CT Scan Platform (Philips, Ohio, USA) (Supplementary Figure S2). The definition of aortic arch was according to the current consensus document for the aortic arch pathologies (11). The status of thrombosis in false lumen was evaluated by delayed phase imaging. The complete thrombosis of the false lumen was defined as no contrast in the interesting area. Postoperatively, all the patients underwent follow-up examination routinely at outpatient and received CTA at 1, 6 months and then annually.

Primary outcomes were defined as technical success, branch patency, thrombosis, and shrinkage of the false lumen or aneurysmal sac. The secondary outcomes were defined as stent graft-related complications and all-cause mortality.

### Statistical Analysis

The presentations of data were determined to be *n* (%), if values are categorical variables and median [interquartile range (IQR)] or mean ± SD, if values are continuous variables. The difference between the groups was compared utilizing the chi-squared test or the Fisher's exact test, if values are categorical variables and the Student's *t*-test, if values are continuous variables. All the statistical analyses were performed using the SPSS software (version 26.0; SPSS Incorporation, Chicago, Illinois, USA). All the tests were 2-sided and *p* < 0.05 was considered to be statistically significant.

## Results

### Feasibility of the Prefenestration

There was no residual stenosis after balloon dilatation of the branch stents before the fatigue test. The MCPF can be easily flared by a balloon at 4–8 atmospheres (Supplementary Figure S1). After the fatigue test, minor residual stenosis was observed from an overhead view (Supplementary Figure S3D).

### Morphological Analysis

**Figure 4:**
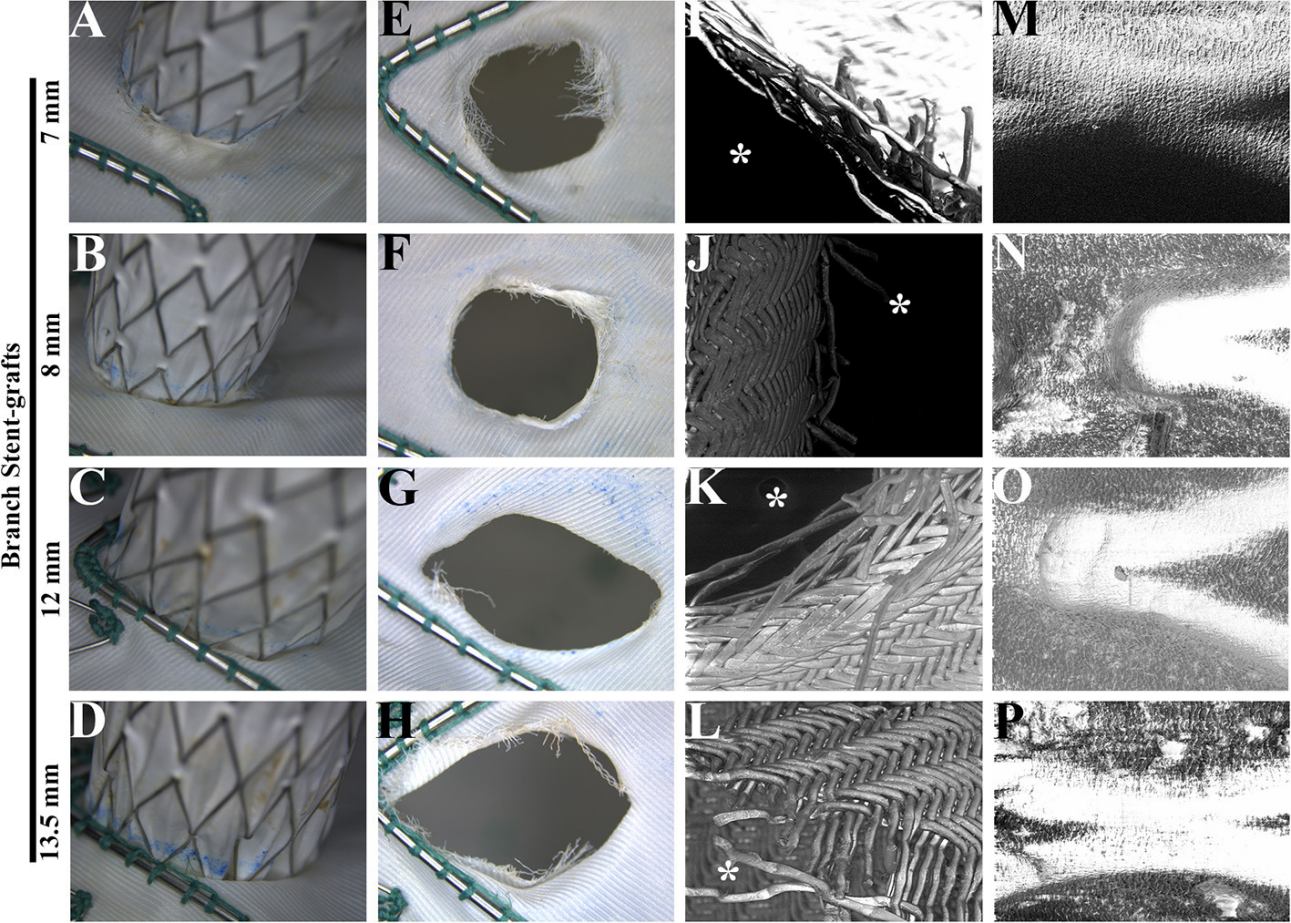
Morphological analysis of main and branch stent grafts after a fatigue test. **(A–D)** The connections of main and branch stent grafts were estimated by light microscopy. **(E–H)** The fabrics around the fenestrations on the main stent grafts were estimated by light microscopy. **(I–L)** The fabrics around the fenestrations on the main stent grafts were estimated by scanning electron microscopy. The magnification was 100×. *, the inner side of fenestrations. **(M–P)** The membrane and metal structures of branch stent grafts were examined by scanning electron microscopy. The magnification was 100×.

After a 5-year simulated cardiac cycle, the structures of the main and branch stents were stable under light microscopy (Figures 4A–D). The fabrics around holes in each group were basically undamaged and remained connected under the light microscopy (Figures 4E–H). Figures 4I–L showed the SEM observation of fabrics around holes of each group, in which the organization was relatively tight. The membrane and metal structures were intact in the branch stents under the observation of SEM (Figures 4M–P).

### Clinical Characteristics

From December 2017 to February 2020, a total of 26 cases (22 males, median age 63 years) were performed with the MCPF technique in our center. Among them, there were 22 (84.6%) cases with hypertension, 1 (3.8%) case with diabetes mellitus (DM), 2 (7.7%) cases with preoperative cerebral infarction, 2 (7.7%) cases with coronary artery diseases (CAD), 1 (3.8%) case with cardiac insufficiency, 1 (3.8%) case with chronic kidney disease (CKD), and 6 (23.1%) cases with chronic obstructive pulmonary disease (COPD) (Table 1).

**Table 1:** Baseline characteristics of patients receiving the MCPF technique.

**Variables**	**Values**
Age, years	63.0 (53.2-69.0)
Male	22 (84.6%)
Smoking	23 (88.5%)
Drinking	15 (57.7%)
Hypertension	22 (84.6%)
DM	1 (3.8%)
Preoperative cerebral infarction	2 (7.7%)
CAD	2 (7.7%)
Cardiac insufficiency	1 (3.8%)
CKD	1 (3.8%)
COPD	6 (23.1%)

*Continuous variables were presented with median [interquartile range (IQR)] and categorical variables were presented with n (%)*.

*MCPF, mini-cross prefenestration; DM, diabetes mellitus; CAD, coronary artery disease; CKD, chronic kidney disease; COPD, chronic occlusive pulmonary disease*.

### Details of Endovascular Procedure

Table 2 demonstrates the details of the endovascular procedure. There were 15 (57.7%) cases of TAD and 11 (42.3%) cases of TAA. There were 8 (30.8%) type I aortic arches, 4 (15.4%) type II aortic arches, and 14 (53.8%) type III aortic arches. The median operation time of the MCPF group was 120.0 (IQR, 95.0–137.5) min. Eighteen (69.2%) patients underwent the reconstruction of the LSCA only. The fenestrations of both the LSCA and LCCA were conducted in 8 (30.8%) patients. The median revascularization time of the LSCA and LCCA was 30.5 (IQR, 22.8–42.0) s and 20.0 (IQR, 18.0–32.0) s separately. The median diameters of the main stent grafts were 34.0 (32.0–34.0) mm and the median oversize rate was 5.5% (IQR, 4.1–12.0%). The median diameters of the LSCA and LCCA branch stents were 10.0 (IQR, 8.0–10.0) mm and 8.0 (IQR, 8.0–9.3) mm. The median lengths of the LSCA and LCCA branch stents were 40.0 (IQR, 40.0–55.0) mm and 60.0 (IQR, 60.0–80.0) mm. No hybrid approach was operated in any case.

**Table 2:** Endovascular procedure details of patients receiving the MCPF technique.

**Variables**	**Values**
Aortic pathology	
TAD	15 (57.7%)
TAA	11 (42.3%)
Arch type	
I	8 (30.8%)
II	4 (15.4%)
III	14 (53.8%)
Operation time, min	120.0 (95.0–137.5)
Details of main stent-grafts	
Oversizing rates of main stent-grafts, %	5.5 (4.1–12.0)
Proximal diameters of main stent-grafts, mm	34.0 (32.0–34.0)
Details of LSCA revascularization (N = 26)	
Revascularization time of LSCA (N = 26), s	30.5 (22.8–42.0)
LSCA stent diameters, mm	10.0 (8.0–10.0)
LSCA stent length, mm	40.0 (40.0–55.0)
Details of LCCA revascularization (N = 8)	
Revascularization time of LCCA (N = 8), s	20.0 (18.0–32.0)
LCCA stent diameters, mm	8.0 (8.0–9.3)
LCCA stent length, mm	60.0 (60.0–80.0)
Hybrid approach	0 (0.0%)

*Continuous variables were presented with median (IQR) and categorical variables were presented with n (%). MCPF, mini-cross prefenestration; TAA, thoracic aortic aneurysm; TAD, thoracic aortic dissection; LSCA, left subclavian artery; LCCA, left common carotid artery*.

### Primary Outcomes

Table 3 demonstrates the primary outcomes of patients receiving the MCPF technique. The postimplantation DSA suggested that no type I/III endoleak or migration was found immediately after the procedure. Furthermore, there were no postoperative MACCE 30 days after TEVAR. The median length of stay was 10.5 (IQR, 8.0–12.5) days.

**Table 3:** Results of patients receiving the MCPF technique.

**Variable**	**Values**
Technique success	26 (100%)
Length of stay, days	10.5 (8.0–12.5)
**30-day events**	0 (0.0%)
Stroke	0 (0.0%)
Endoleak	
Type I	0 (0.0%)
Type II	0 (0.0%)
Type III	0 (0.0%)
Retrograde AAD	0 (0.0%)
New dissection	0 (0.0%)
Rupture	0 (0.0%)
Patency of branch	
LCCA	8 (100%)
LSCA	26 (100%)
All-cause mortality	0 (0.0%)
**Events at last follow-up**	0 (0.0%)
Stroke	0 (0.0%)
Endoleak	
sType I	0 (0.0%)
Type II	0 (0.0%)
Type III	0 (0.0%)
Retrograde AAD	1 (3.8%)
New dissection	0 (0.0%)
Rupture	0 (0.0%)
Patency of branch	26 (100%)
LCCA	8 (100%)
LSCA	26 (100%)
All-cause mortality	0 (0.0%)

*Values were presented with n (%)*.

*MCPF, mini-cross prefenestration; retrograde AAD, retrograde type A aortic dissection; LCCA, left common carotid artery; LSCA, left subclavian artery*.

In the first 30 days after TEVAR, there were no adverse clinical outcomes occurring (Table 3). The 1-month CTA imaging indicated that no type I, II, or III endoleak, retrograde AAD, new dissection, or rupture was observed. The patency rates of LCCA and LCSA 30 days after TEVAR remained to be 100%.

**Figure 5:**
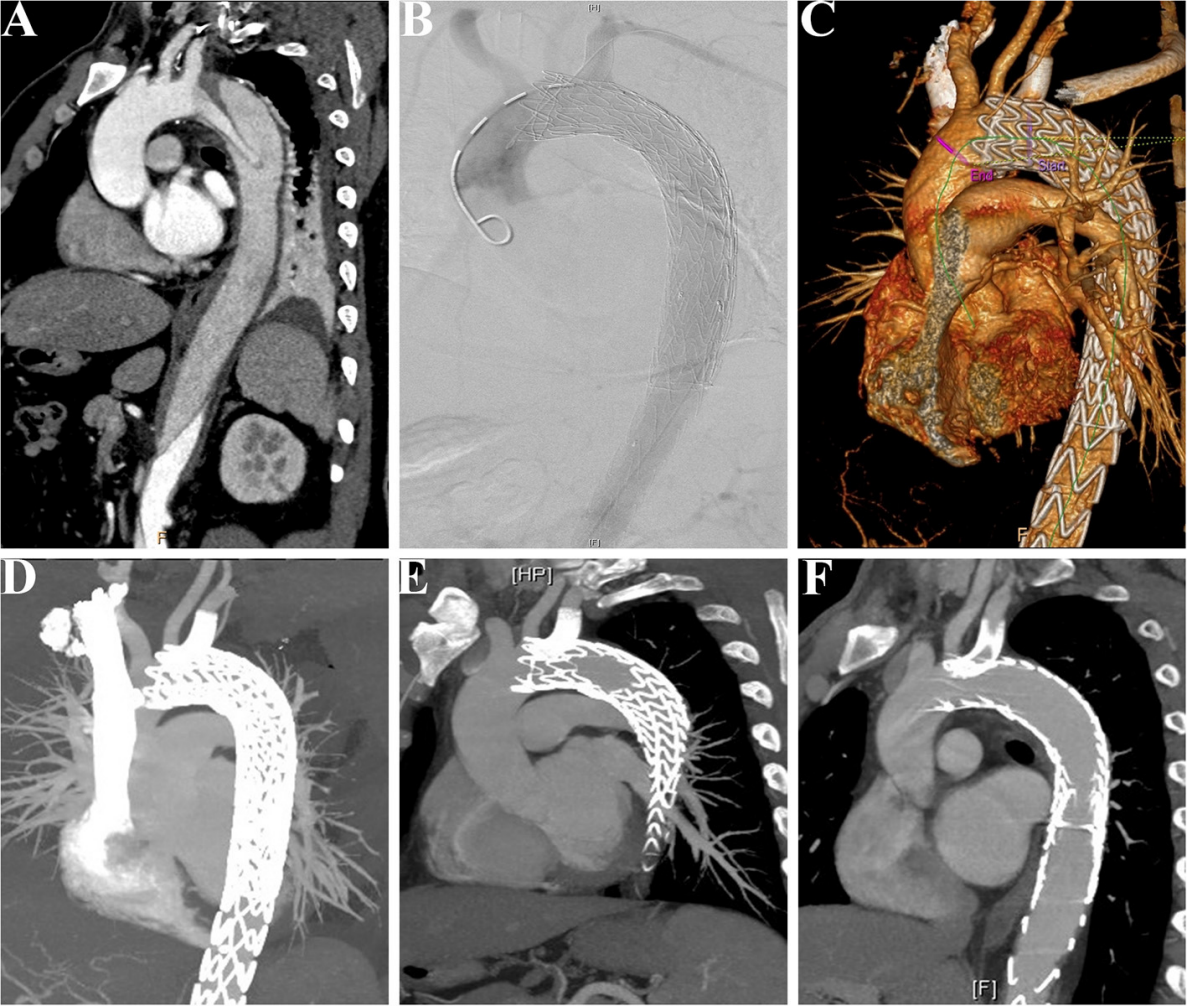
Patency of the LSCA was followed-up in a patient receiving the MCPF technique. **(A)** Preoperative CTA showed that a patient suffered from thoracic aortic dissection, which involved the LSCA. **(B)** Intraoperative aortogram demonstrated that the LSCA was revascularized and false lumen was excluded. **(C)** Before discharge, CTA was conducted to confirm the patency of the LSCA and exclusion of false lumen. **(D)** CTA at follow-up of 6 months. **(E)** CTA at follow-up of 12 months. **(F)** CTA at follow-up of 24 months. LSCA, left subclavian artery; MCPF, mini-cross prefenestration; CTA, CT angiography.

During the median follow-up duration of 38.9 (range, 18.8–44.2) months, there was one (3.8%) case of retrograde type A aortic dissection (AAD) at 3 months after TEVAR. The patient then received the total arch replacement and survived until the last follow-up. From the investigation of follow-up CTA, all the branch stents were patent (Figure 5) and had no other stent graft-related complications (Table 3). There was no all-cause mortality during the follow-up.

### Aortic Remodeling After the MCPF Procedure

**Figure 6:**
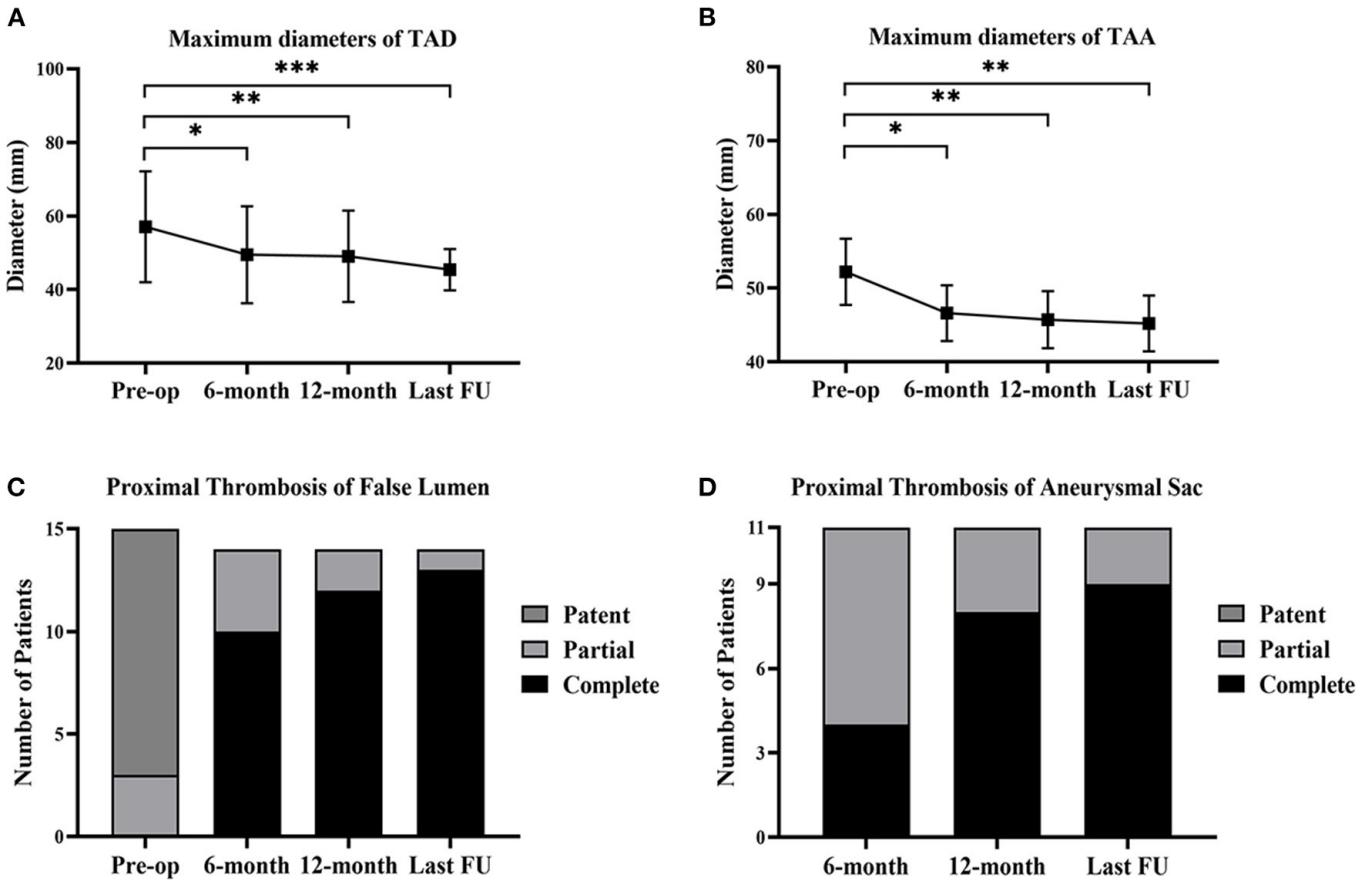
Aortic remodeling and proximal thrombosis of false lumen/aneurysmal sac at follow-up. **(A)** The changes of maximum diameters of TAD before the operation, at 6-month, 12-month, and the last follow-up. **(B)** The changes of maximum diameters of TAA before the operation, at 6-month, 12-month, and the last follow-up. **(C)** The proximal thrombosis of false lumen in TAD (*n* = 15) before the operation, at 6-month, 12-month, and the last follow-up. One patient suffered from retrograde type A aortic dissection after 3 months and received the total arch replacement. **(D)** The proximal thrombosis of aneurysmal sac in TAA (*n* = 11) at 6-month, 12-month, and the last follow-up. *p < 0.05; **p < 0.01; ***p < 0.001. TAD, thoracic aortic dissection; TAA, thoracic aortic aneurysm; FU, follow-up.

Compared with the preoperative conditions, the maximum aortic diameters were significantly decreased after the 6-month follow-up and remained stable at the last follow-up (Figures 6A,B). After implantation, the proximal complete thrombosis of the false lumen in patients with TAD was 10 (71.4%), 12 (85.7%), and 13 (92.9%) at the time of 6, 12 months, and the last follow-up (Figure 6C). The prevalence of complete thrombosis in aneurysmal sac increased from 4 (36.4%) at 6-month follow-up to 9 (81.8%) at the last follow-up (Figure 6D).

## Discussion

With the development of endovascular devices and techniques, mortality and morbidity were significantly decreased in patients with TAA and TAD after TEVAR (12). A *post-hoc* analysis of the Study of Thoracic Aortic Type B Dissection Using Endoluminal Repair (STABLE) trial I and II found that decreased proximal seal length was related with stent graft-related adverse events (13). When considering the LSCA involved aortic diseases, a 20-mm proximal seal length was recommended by a previous study (3). In this background, the coverage rate of the LSCA was 26–40% for the adequate proximal landing zone (14, 15).

However, the prevalence of stroke was confirmed to be lower in the condition of uncoverage or revascularization of the LSCA than that of complete coverage (2.2–5.3 vs. 8.0–9.1%) (16, 17). Thus, the LSCA was recommended to be preserved for the concerns with severe limb ischemia and stroke (18, 19). For the purpose of rapid branch artery revascularization, a novel prefenestration technique with existing devices was conceived and confirmed by *in vitro* and clinical experiments in our center.

Generally, there were three advantages to this technique as follows:

First, the location of the fenestration was confirmed by the traction guidewire (Figure 3B), which simultaneously reduced the endovascular procedure time and risk of dislocation between the branch artery and fenestration. This design was inspired from the construction of single-branched Castor stent graft, which is confirmed to be effective and safe for aortic arch pathologies (20). The guidewire preloaded in the main stent graft could help the operator to easily place the stent graft in the designed location (Figure 3B, white arrows). In the previous study, a radiopaque marker was always needed for the right implantation of stent grafts (21). However, plenty of time used for markers increased the burden of patients. According to the previous studies about handmade fenestration for the LSCA, the mean operation time was 171–176 min, which was significantly longer than our data (mean operation time, 125 min) (22, 23).

Second, the revascularization of the LSCA/LCCA was literally rapid around 60 s in this study (Figure 3D; Table 2). Given the importance of supra-arch vessels, the rapid LSCA/LCCA revascularization is demanding study (24). In this study, the prefenestration had a 5 × 5 mm hole, which allowed the blood flow to come through. More importantly, when a main stent graft was implanted, the balloon was already prepared for rapid revascularization (Figure 3C). Although the traditional fenestration technique does not need to consider the time of revascularization, unsuitable fenestration may cause unexpected coverage or endoleak (25). In terms of the *in-situ* fenestration technique, it takes time to penetrate the main stent grafts, which may increase the risk of cerebral infarction (24, 26).

Third, a 5 × 5 mm prefenestration on Valiant Stent Grafts then engaged with stent grafts was confirmed safety and integrity in a 5-year stimulated fatigue test and a median 38.9-month clinical follow-up. Although *in-vitro* experiment showed that a laser or needle effectively generated a hole in the membrane, the controllability was severely affected by the aortic pulsation *in vivo* (24, 27). The laser may cause serious damage to the membrane, which resulted in junction weakness and gutter leakage (27). In the fatigue test of this study, no obvious damage was found beyond the fenestration at 262,800,000 cardiac cycles. There was also no disconnection or dislocation between the main and branch stents found in the follow-up CTA. In summary, the MCPF might be more controllable *in vivo* and safer compared with the current *in situ* fenestration technique.

## Conclusion

The existing evidence demonstrated the safety, rapid branch artery revascularization, and positive aortic remodeling of the novel technique. Long-term observation is demanded to prove the durability.

## Data Availability Statement

The raw data supporting the conclusions of this article will be made available by the authors, without undue reservation.

## Ethics Statement

This retrospective study was approved by the Institutional Review Board of Shanghai Changhai Hospital (ClinicalTrials.gov Identifier: NCT04544579). All patients signed the informed consent. The patients/participants provided their written informed consent to participate in this study.

## Author Contributions

YP contributes to the preclinical and clinical administrator. HZ and YX contribute to the investigation and writing. JZ contributes to the writing, review, editing, and supervision. ZJ contributes to the conceptualization and project administration. All the authors have read and approved the final version of the manuscript.

## Funding

This study, collection, analysis, interpretation of data, and preparation of the manuscript were supported by the source of funding as follows: the National Natural Science Foundation of China (81870366), the Science and Technology Innovation Action Plan in Shanghai (20JC1418700), and the Dawn Project of Shanghai (19SG31).

## Conflict of Interest

The authors declare that the research was conducted in the absence of any commercial or financial relationships that could be construed as a potential conflict of interest.

## Publisher's Note

All claims expressed in this article are solely those of the authors and do not necessarily represent those of their affiliated organizations, or those of the publisher, the editors and the reviewers. Any product that may be evaluated in this article, or claim that may be made by its manufacturer, is not guaranteed or endorsed by the publisher.

## Supplementary Material

Supplementary Figure S1 The demonstration of the balloon water pressure in different diameters of branch stent grafts. The mini-fenestration could be completely flared by a balloon at 4 atmospheres in diameter of 7 mm **(A)**, 5.5 atmospheres in diameter of 8 mm **(B)**, 8 atmospheres in diameter of 12 mm **(C)** and 6 atmospheres in diameter of 13.5 mm **(D)**.

Supplementary Figure S2 The evaluation and measurement in a CTA scan platform. The measurement of aortic diameters. All the proximal thrombosis and measurement were performed by an experienced radiologist with the Brilliance CT Scan Platform (Philips, Ohio, USA). **(A)** The aortic arch determination was made by 3-dimensional construction of CTA. **(B)** The proximal thrombosis evaluation. The green arrow showed the LSCA. The red arrow showed the patent false lumen of TAD. The white arrow showed the true lumen of TAD. **(C)** The measurement of maximum descending aortic diameter. The black arrow showed the ascending aorta. The yellow arrow showed the descending dissecting aorta. CTA, CT angiography; LSCA, left subclavian artery; TAD, thoracic aortic dissection.

Supplementary Figure S3 The process of *in-vitro* stent grafts removal and naked-eye evaluation. **(A)** After the fatigue test, the silicone models were disassembled from the machine and cut along the longitudinal axis. **(B)** The stent grafts were removed from silicone models. **(C)** There was no obvious disconnection between the main and branch stent grafts by naked eyes. **(D)** There was no obvious deformation in the branch stent graft from the top view.

Supplementary Movie 1 The prefenestrations were made on the Valiant Captivia Stent Graft with a 5 mm × 5mm cross.

Supplementary Movie 2 Before the stent graft was reloaded into the delivery system, the tip of a guidewire (RF^*^GA35153M, Terumo, Japan) was induced through the fenestration for the purpose of traction from the aorta to the LSCA. LSCA, left subclavian artery.
