# Supplementary figures and images for: The Mini-Cross Prefenestration for Endovascular Repair of Aortic Arch Pathologies
Source: Front Cardiovasc Med. 2022 Jan 11;8:745871. doi: 10.3389/fcvm.2021.745871 (PMC8787069; doi:10.3389/fcvm.2021.745871)

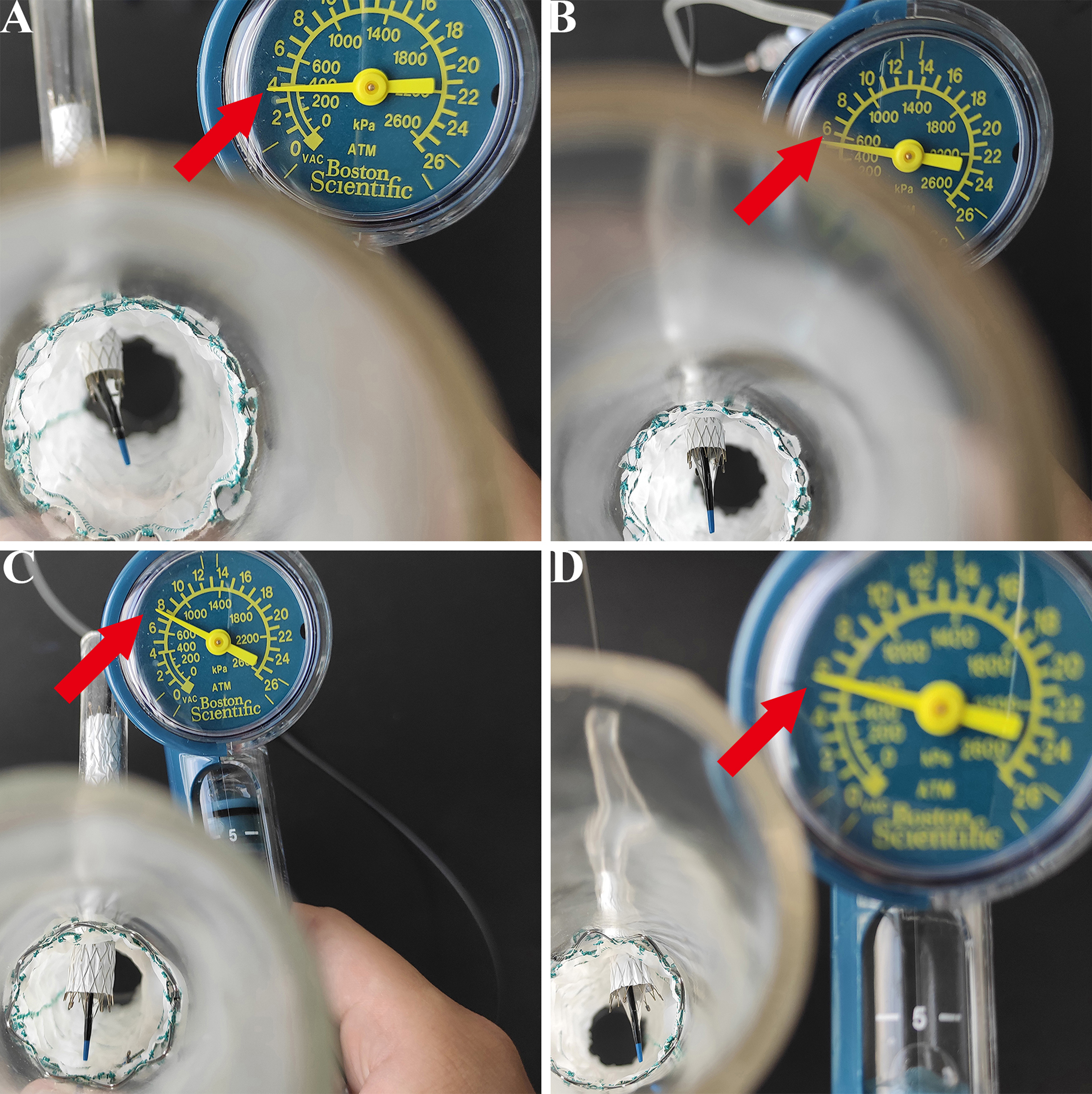

Supplement: Supplementary Figure S1 — The demonstration of the balloon water pressure in different diameters of branch stent grafts. The mini-fenestration could be completely flared by a balloon at 4 atmospheres in diameter of 7 mm (A), 5.5 atmospheres in diameter of 8 mm (B), 8 atmospheres in diameter of 12 mm (C) and 6 atmospheres in diameter of 13.5 mm (D). [file Image_1.JPEG]

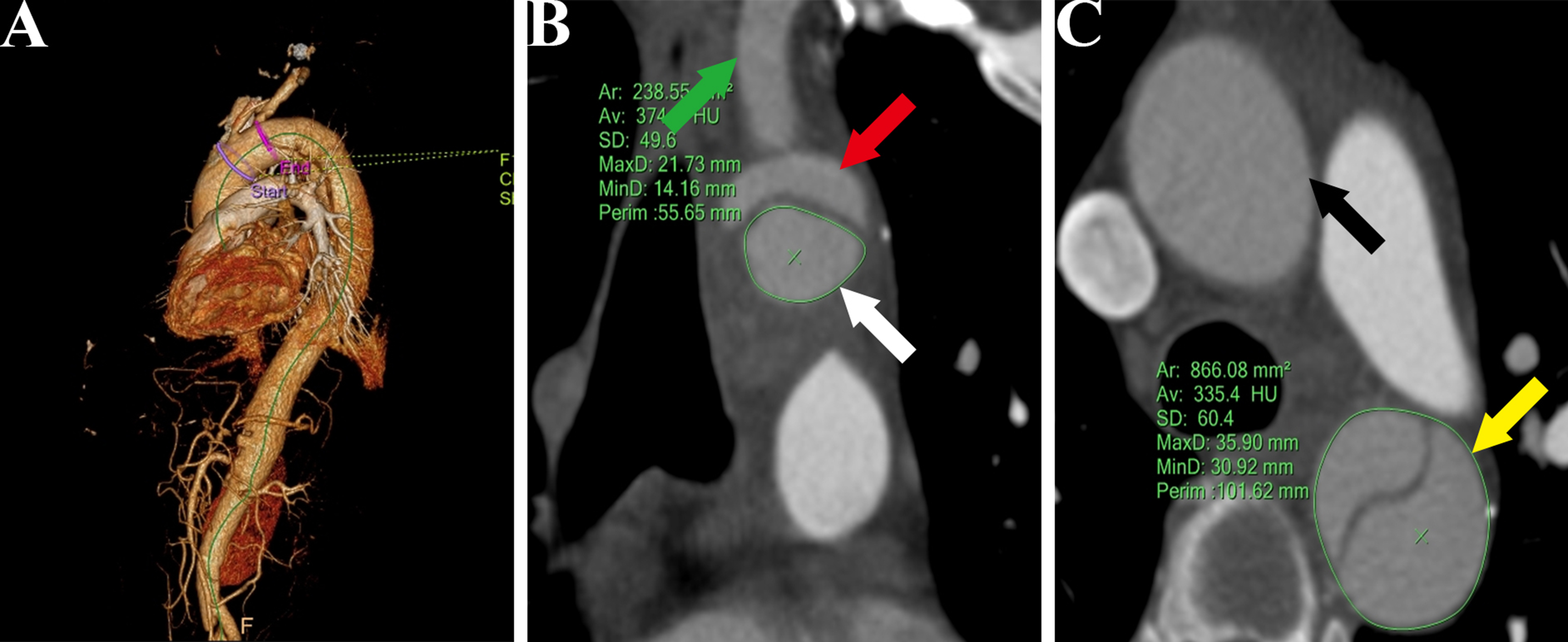

Supplement: Supplementary Figure S2 — The evaluation and measurement in a CTA scan platform. The measurement of aortic diameters. All the proximal thrombosis and measurement were performed by an experienced radiologist with the Brilliance CT Scan Platform (Philips, Ohio, USA). (A) The aortic arch determination was made by 3-dimensional construction of CTA. (B) The proximal thrombosis evaluation. The green arrow showed the LSCA. The red arrow showed the patent false lumen of TAD. The white arrow showed the true lumen of TAD. (C) The measurement of maximum descending aortic diameter. The black arrow showed the ascending aorta. The yellow arrow showed the descending dissecting aorta. CTA, CT angiography; LSCA, left subclavian artery; TAD, thoracic aortic dissection. [file Image_2.JPEG]

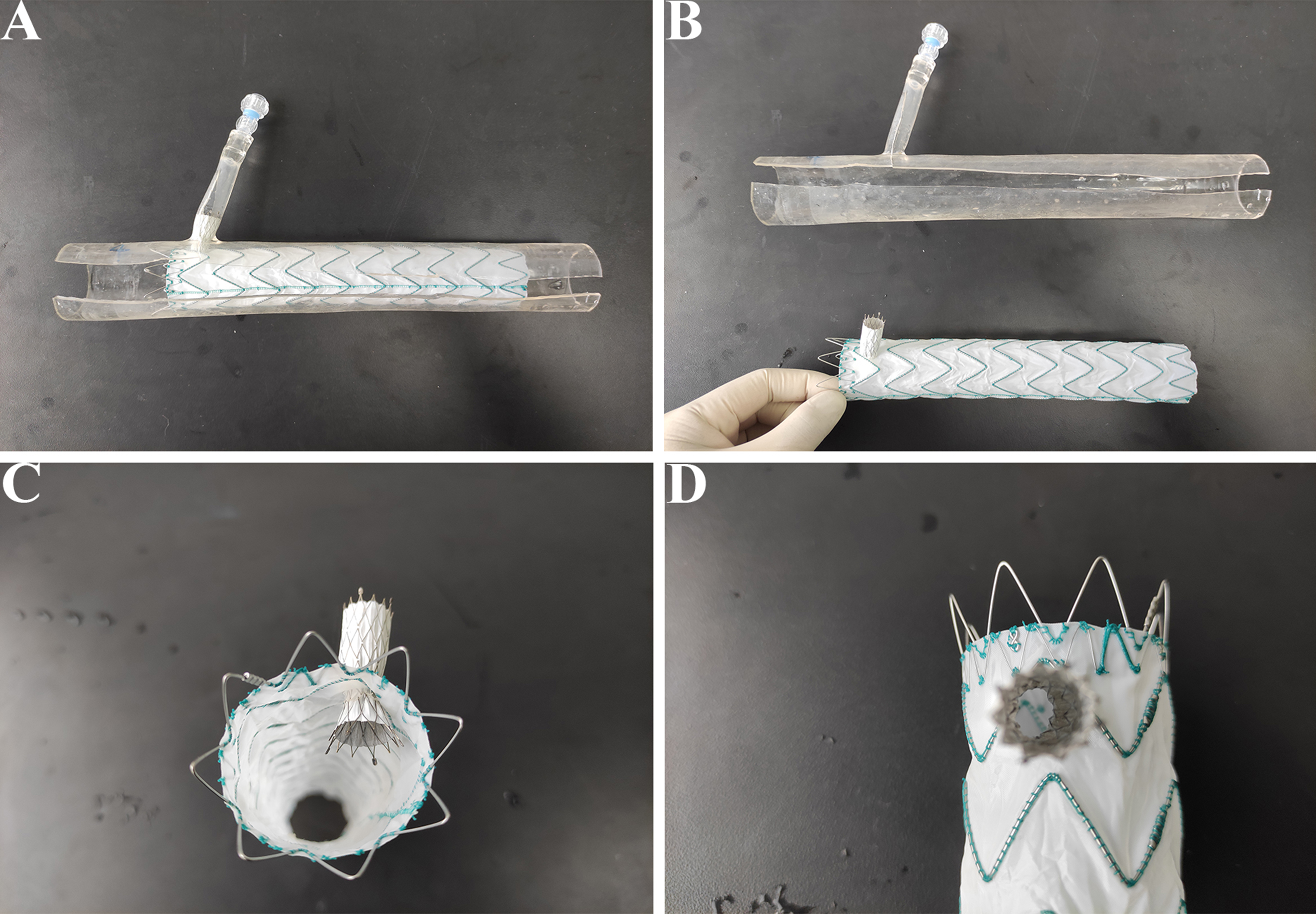

Supplement: Supplementary Figure S3 — The process of in-vitro stent grafts removal and naked-eye evaluation. (A) After the fatigue test, the silicone models were disassembled from the machine and cut along the longitudinal axis. (B) The stent grafts were removed from silicone models. (C) There was no obvious disconnection between the main and branch stent grafts by naked eyes. (D) There was no obvious deformation in the branch stent graft from the top view. [file Image_3.JPEG]
